# Effect of Colesevelam HCl Monotherapy on Lipid Particles in Type 2 Diabetes Mellitus

**DOI:** 10.1007/s10557-014-6516-y

**Published:** 2014-04-08

**Authors:** Robert S. Rosenson, Scott P. Rigby, Michael R. Jones, Hubert S. Chou

**Affiliations:** 1 Cardiometabolic Disorders, Mt. Sinai Heart, Icahn School of Medicine at Mount Sinai, 1425 Madison Avenue, MC1 Level, New York, NY 10029 USA; 2 Summit Research Group, 4466 Darrow Road, Building B, Suite 5, Stow, OH 44224 USA; 3 Unity Health Network, LLC, 307 W. Main Street, Suite B, Kent, OH 44240 USA; 4 Daiichi Sankyo, Inc., 2 Hilton Court, Parsippany, NJ 07054 USA; 5 Daiichi Sankyo Pharma Development, 399 Thornall Street, Edison, NJ 08837 USA; 6 Present Address: Healthcare Consultant, 9156 Pumpkin Ridge, Port St. Lucie, FL 34986 USA

**Keywords:** Colesevelam, Monotherapy, Lipoprotein particles, Low-density lipoprotein, Nuclear magnetic resonance, Type 2 diabetes mellitus

## Abstract

**Purpose:**

In addition to lowering hemoglobin A1C, colesevelam has been shown to improve the atherogenic lipoprotein profile of subjects with type 2 diabetes mellitus (T2DM) when used in combination with metformin and/or sulfonylureas. A recent study evaluated the effects of colesevelam as antidiabetes monotherapy in adults with T2DM who had inadequate glycemic control (hemoglobin A1C ≥7.5 to ≤9.5 %) with diet and exercise alone; we report here the effects on lipoprotein particle subclasses.

**Methods:**

Subjects were randomized to receive oral colesevelam 3.75 g/day (*n* = 176) or placebo (*n* = 181) for 24 weeks. Changes in lipoprotein particle subclasses were determined by nuclear magnetic resonance spectroscopy.

**Results:**

At Week 24 with last observation carried forward, colesevelam produced a reduction in total low-density lipoprotein (LDL) particle concentration (baseline: 1,611 nmol/L; least-squares [LS] mean treatment difference: −143 nmol/L, *p* < 0.0001) versus placebo; reductions were also seen in large, small, and very small LDL particle concentrations (all *p* < 0.05). There was also a reduction in total very low-density lipoprotein (VLDL) and chylomicron particle concentration (baseline: 88 nmol/L; LS mean treatment difference: −1 nmol/L, *p* = 0.82) that resulted from a lowering in small VLDL particle concentration (baseline: 45 nmol/L; LS mean treatment difference: −5 nmol/L, *p* = 0.03). In addition, with colesevelam there was an increase in total high-density lipoprotein (HDL) particle concentration versus placebo (baseline: 31 μmol/L; LS mean treatment difference: +0.6 μmol/L, *p* = 0.20), due to increases in the large (baseline: 5 μmol/L; LS mean treatment difference: +0.5 μmol/L, *p* = 0.007) and medium (baseline: 3 μmol/L; LS mean treatment difference: +0.8 μmol/L, *p* = 0.02) HDL subclasses.

**Conclusions:**

Colesevelam monotherapy in subjects with T2DM resulted in generally favorable changes in certain lipoprotein subclass profiles compared with placebo.

## Introduction

Insulin resistance and type 2 diabetes mellitus (T2DM) are associated with a variety of lipid and lipoprotein abnormalities, including reduced high-density lipoprotein (HDL) cholesterol levels, elevated triglyceride levels, and an altered distribution of lipoprotein particles [1]. Patients with insulin resistance also have alterations to the lipoprotein subclass profile that are not reflected by standard lipid panels [2, 3]. In particular, very low-density lipoprotein (VLDL) particle concentration (VLDL-P) and low-density lipoprotein (LDL) particle concentration (LDL-P) are increased and HDL particle concentration (HDL-P) is decreased in patients with insulin resistance or T2DM compared with individuals who have normal insulin sensitivity [2]. Among the lipoprotein subclasses, VLDL particle sizes are increased and LDL and HDL particle sizes are decreased in patients with insulin resistance or T2DM. These effects are seen despite normal or near-normal levels of LDL cholesterol and total cholesterol [3].

This altered distribution of lipoprotein particles (particularly LDL particles) is relevant to cardiovascular disease (CVD) risk [4]. LDL-P is predictive for coronary endothelial dysfunction and CVD risk [5, 6] and may be a better predictor than LDL cholesterol, especially in patients with insulin resistance or T2DM [4, 7–9]. In addition, HDL-P is inversely correlated with carotid intima-media thickness and coronary heart disease risk [10–12]. Consequently, reducing total LDL-P through changes in the distribution of lipoprotein particles in patients with hypercholesterolemia via intervention with lipid-lowering agents is a potentially good therapeutic strategy to reduce CVD risk [13]. However, it should be noted that although clinical data exist demonstrating the ability of some cholesterol-lowering drugs to improve the LDL subclass distribution profile, the supporting data come from predominately smaller studies. Thus, the ability of these agents to reduce vascular risk still needs to be demonstrated in large clinical trials. For example, it is important to determine the clinical benefit of drug regimens that reduce small LDL particle fractions versus those that reduce all fractions simultaneously, as well as to identify the subclass distribution profile produced by each lipid-lowering agent, so that an agent may be chosen to appropriately modify the lipid profile of the individual patient [14]. In addition, determining which agents increase HDL-P is also important, as HDL-P is inversely correlated with carotid intima-media thickness and cardiovascular events, and therefore administering an agent that increases HDL-P may help to further reduce CVD risk [10–12, 15].

The bile acid sequestrant colesevelam has been shown to improve glycemic and lipid parameters in subjects with T2DM when added to metformin-, sulfonylurea-, or insulin-based therapy [16–19]. In addition, treatment with colesevelam in combination with metformin and/or sulfonylureas has been shown to result in a more beneficial lipoprotein profile in subjects with T2DM [20, 21]. A recent study evaluated the effects of colesevelam as an antidiabetes monotherapy in adults with T2DM who were inadequately controlled by diet and exercise alone (ClinicalTrials.gov identifier: NCT00789737) [22]. Here we report the effects of colesevelam monotherapy on the concentrations and sizes of various lipoprotein particle subclasses from this study.

## Materials and Methods

### Study Design

This was a 24-week, randomized, double-blind, placebo-controlled, parallel-group study conducted at approximately 80 study centers in the United States. The study was conducted in compliance with independent ethics committee/institutional review board regulations, good clinical practice guidelines, and the principles of the Declaration of Helsinki. Institutional review board approval was obtained before initiation of the study, and all subjects provided written informed consent.

Details of this study, including design, inclusion/exclusion criteria, and statistical methods, have been described previously [22]. Briefly, the study enrolled subjects aged ≥18 years with a diagnosis of T2DM (based on American Diabetes Association diagnostic criteria) who were untreated at the time of screening (i.e. treatment-naïve or not receiving antihyperglycemic medication within ≥3 months prior to screening). Subjects were required to have a hemoglobin A1C (A1C) level ≥7.5 % and ≤9.5 % at screening, a fasting plasma glucose (FPG) level ≤240 mg/dL at randomization, and a fasting C-peptide level >0.5 ng/mL at screening. Subjects were not randomized if they had persistent FPG levels >240 mg/dL during the placebo lead-in period.

Following a 2-week single-blind placebo lead-in period, subjects were randomized to receive oral colesevelam 3.75 g/day (6 × 625 mg tablets) or placebo for 24 weeks. Subjects taking concomitant medications must have been at stable doses for ≥30 days prior to enrollment and not have been anticipated to require adjustment during the study period. To the extent possible, subjects were instructed to maintain the same dosages of established lipid-lowering agents throughout the double-blind treatment period to minimize confounding the evaluation of the lipid-lowering effects of colesevelam versus placebo.

### Assessments

Scheduled clinic visits were at Weeks 0, 4, 8, 16, and 24 after an overnight fast. Samples for chemistry, hematology, A1C, and FPG were collected at screening and every visit from baseline (Week 0) to the end of treatment (Week 24). The primary efficacy variable was A1C level at Week 24. Secondary efficacy variables included FPG, high-sensitivity C-reactive protein, total cholesterol, LDL cholesterol, HDL cholesterol, non-HDL cholesterol, triglyceride, apolipoprotein A-I (apoA-I), and apoB levels. A standard meal tolerance test was conducted at baseline and at Week 24 to evaluate the glucose excursion. Fasting blood samples were obtained for the assessment of lipid particle parameters at baseline and Week 24. Particle parameters were determined by nuclear magnetic resonance (NMR) spectroscopy [23]. In this technique, distributions and concentrations of individual lipid particle subclasses are determined based on the fact that lipoprotein particles within a specified diameter range emit a distinctive magnetic resonance signal with its signal intensity proportional to lipid mass concentration [14, 24]. This analysis evaluated changes in lipid particle parameters (mean lipoprotein particle concentrations and sizes) from baseline to Week 24.

### Statistics

The intention-to-treat (ITT) population was the primary population for analysis, defined as all randomized subjects who received ≥1 dose of study medication and had an A1C or FPG measurement at baseline and ≥1 post-baseline measurement prior to taking any antihyperglycemic rescue medication. The analysis compared changes from baseline between colesevelam and placebo to rescue or Week 24 with last observation carried forward (LOCF) using the ITT population. Between-group differences were evaluated using an analysis of covariance model, with treatment as a fixed effect and baseline as a covariate; all statistical tests were 2-sided at a 5 % significance level. Because of the low number of subjects on HMG-CoA reductase inhibitors (statins), the results were not analyzed separately according to those subjects taking both a statin and colesevelam versus colesevelam without a statin.

## Results

### Subjects

A total of 357 subjects were randomized to receive colesevelam (*n* = 176) or placebo (*n* = 181); the ITT population comprised 344 subjects (175 and 169, respectively). Demographic and baseline characteristics of all randomized subjects were generally similar between treatment groups, as summarized in Table 1. The mean age was 52.2 years and a high percentage of subjects were Hispanic/Latino (46.5 %). The mean duration of T2DM was 4.1 years and the mean body mass index was 31.9 kg/m^2^. Overall, 14.2 % of subjects in the ITT population were taking statins and 2.9 % were taking fibrates. Mean changes in weight from baseline to end of study/early termination were similar for subjects in both the colesevelam and placebo groups (−0.58 and −0.70 kg, respectively).

**Table 1:** Demographic and baseline characteristics (all randomized subjects)

Characteristic^a^	Colesevelam (*n* = 176)	Placebo (*n* = 181)
Age (years), mean (SD)	52.6 (10.25)	51.8 (10.52)
Sex, *n* (%)		
Male	94 (53.4)	89 (49.2)
Race, *n* (%)		
Caucasian	122 (69.3)	131 (72.4)
Black	27 (15.3)	29 (16.0)
Asian	12 (6.8)	8 (4.4)
American Indian/Alaskan native	12 (6.8)	11 (6.1)
Native Hawaiian/Pacific Islander	1 (0.6)	0 (0.0)
Other	2 (1.1)	2 (1.1)
Ethnicity, *n* (%)		
Hispanic/Latino	86 (48.9)	80 (44.2)
Not Hispanic/Latino	90 (51.1)	101 (55.8)
Body mass index (kg/m^2^), mean (SD)	32.0 (6.50)	31.8 (4.94)
Hemoglobin A1C (%), mean (SD)	8.25 (0.684)	8.18 (0.697)
Fasting plasma glucose, mg/dL, mean (SD)	172.5 (46.44)	168.0 (37.61)
Fasting insulin, μIU/mL, mean (SD)	17.7 (16.63)	17.9 (14.04)
Duration of type 2 diabetes (years), mean (SD)	4.3 (4.69)	3.9 (4.39)
Total cholesterol (mg/dL), mean (SD)	202.9 (39.69)	200.6 (40.37)
LDL cholesterol (mg/dL), mean (SD)	122.5 (33.80)	119.0 (33.17)
HDL cholesterol (mg/dL), median (range)	43.0 (23.0–84.5)	43.5 (24.0–100.0)
Non-HDL cholesterol (mg/dL), mean (SD)	159.4 (38.39)	155.8 (39.83)
Triglycerides (mg/dL), median (range)	162.3 (49.0–675.0)	169.0 (48.0–680.0)
Apolipoprotein A-I (mg/dL), mean (SD)	145.8 (21.62)	146.6 (23.99)
Apolipoprotein B (mg/dL), mean (SD)	114.0 (25.99)	113.7 (28.06)

*HDL* high-density lipoprotein, *LDL* low-density lipoprotein, *SD* standard deviation

^a^There were no statistically significant between-group differences for any demographic variable or baseline variable

### Efficacy

From baseline to Week 24, treatment with colesevelam, compared with placebo, resulted in significant least-squares (LS) mean reductions in A1C (treatment difference: −0.3 %; *p* = 0.01; primary efficacy variable), LDL cholesterol (−13.6 mg/dL; *p* < 0.0001), total cholesterol (−9.8 mg/dL; *p* = 0.0017), non-HDL cholesterol (−11.1 mg/dL; *p* = 0.0004), and apoB levels (−7.0 mg/dL; *p* = 0.0002). A significant median increase in triglycerides (treatment difference: 16.5 mg/dL; *p* < 0.05) and LS mean increase in apoA-I levels (treatment difference: 3.4 mg/dL; *p* < 0.05) was also observed with colesevelam compared with placebo [22]. The percent changes in lipid parameters from baseline to Week 24 are shown in Table 2. Colesevelam resulted in statistically significant mean percent reductions in total cholesterol, LDL-C, non-HDL-C, apoB, and apoA-I levels, and a statistically significant median percent increase in triglycerides, compared with placebo.

**Table 2:** Percent change in lipid parameters

Parameter	*N*	Percent change from baseline		
			Treatment difference	
		LS mean	LS mean	*p*-value
Total cholesterol, mg/dL				
Placebo	160	2	−5	<0.001
Colesevelam	164	−3		
LDL-C, mg/dL				
Placebo	160	1	−11	<0.0001
Colesevelam	162	−10		
HDL-C, mg/dL				
Placebo	160	−0.1	2	0.31
Colesevelam	164	2		
Non-HDL-C, mg/dL				
Placebo	160	3	−7	<0.001
Colesevelam	164	−4		
Triglyceride, mg/dL^a^				
Placebo	160	6	10	0.03
Colesevelam	164	15		
apoA-I, mg/dL				
Placebo	160	0.9	2	0.04
Colesevelam	164	3		
apoB, mg/dL				
Placebo	160	0.9	−6	<0.001
Colesevelam	164	−6		

Reported changes are measured from baseline to Week 24 with last observation carried forward (intent-to-treat population)

*apoA-I* apolipoprotein A-I, *apo B* apolipoprotein B, *HDL-C* high-density lipoprotein cholesterol, *LDL-C* low-density lipoprotein cholesterol, *LS* least squares

^a^Parameter is not normally distributed. The median value is reported rather than the mean value

#### Nuclear Magnetic Resonance Findings

**Fig. 1:**
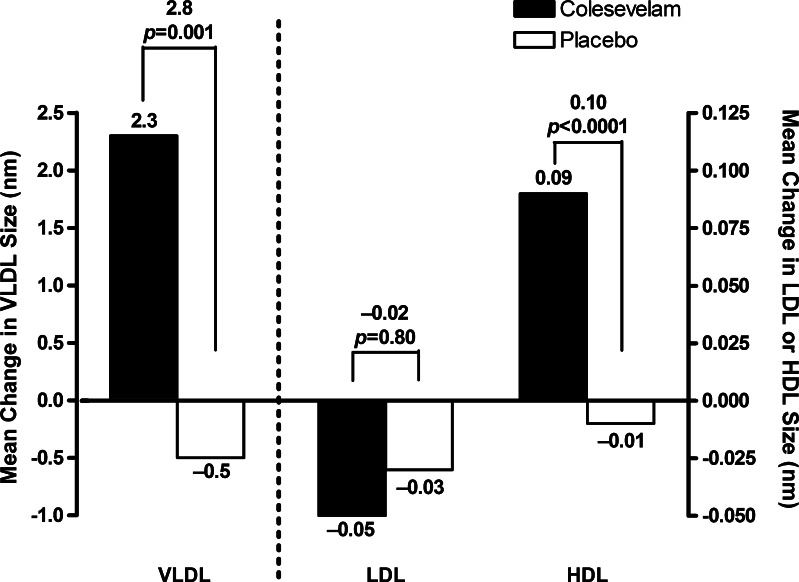
Least-squares mean change in lipid particle size, from baseline to Week 24, with last observation carried forward. *HDL* high-density lipoprotein, *LDL* low-density lipoprotein, *VLDL* very low-density lipoprotein

Subjects who received colesevelam, compared with placebo, had significant reductions in total LDL-P (baseline: 1,611 nmol/L; LS mean treatment difference: −143 nmol/L; *p* < 0.0001), as well as large (baseline: 339 nmol/L; −60 nmol/L; *p* = 0.002), small (baseline: 1,203 nmol/L; −82 nmol/L; *p* < 0.05), and very small LDL-P (baseline: 963 nmol/L; −73 nmol/L; *p* = 0.03; Table 3). Although the subjects who received colesevelam had both improvements in glycemic control and reductions in LDL-P, post-hoc correlation analysis showed no significant correlations between changes in LDL-P and changes in A1C or FPG. Change in LDL particle size was not significantly different between the treatment groups (Fig. 1).

**Table 3:** Change in lipid particle concentrations

Parameter	Baseline	Endpoint	Change from baseline		
				Treatment difference	
			LS mean	LS mean	*p*-value^a^
Total LDL-P, nmol/L					
Placebo	1,563	1,591	24	−143	<0.0001
Colesevelam	1,611	1,487	−119		
Large LDL-P, nmol/L					
Placebo	325	328	2	−60	0.002
Colesevelam	339	278	−59		
Medium small LDL-P, nmol/L					
Placebo	236	240	3	−9	0.31
Colesevelam	240	234	−6		
Small LDL-P, nmol/L					
Placebo	1,169	1,195	22	−82	<0.05
Colesevelam	1,203	1,140	−60		
Very small LDL-P, nmol/L					
Placebo	933	955	19	−73	0.03
Colesevelam	963	906	−54		
Total VLDL-P/chylomicron, nmol/L					
Placebo	86	92	6	−0.8	0.82
Colesevelam	88	94	5		
Large VLDL/chylomicron, nmol/L					
Placebo	6	7	0.7	1	0.08
Colesevelam	6	8	2		
Medium VLDL, nmol/L					
Placebo	38	41	4	4	0.17
Colesevelam	38	45	7		
Small VLDL, nmol/L					
Placebo	42	44	2	−5	0.03
Colesevelam	45	41	−3		
Total HDL-P, umol/L					
Placebo	31	32	0.4	0.6	0.20
Colesevelam	31	32	1		
Large HDL-P, umol/L					
Placebo	5	5	−0.2	0.5	0.007
Colesevelam	5	5	4		
Medium HDL-P, umol/L					
Placebo	3	3	0.2	0.8	0.02
Colesevelam	3	4	1		
Small HDL-P, umol/L					
Placebo	24	24	0.3	−0.8	0.10
Colesevelam	24	23	−0.5		

Reported changes are measured from baseline to Week 24 with last observation carried forward (intent-to-treat population)

*HDL-P* high-density lipoprotein particle concentration, *LDL-P* low-density lipoprotein particle concentration, *LS* least squares, *VLDL-P* very low-density lipoprotein particle concentration

^a^ *p*-values are not adjusted for multiple comparisons

Subjects in the colesevelam group, compared with the placebo group, had a nonsignificant reduction in total VLDL and chylomicron particle concentration (LS mean treatment difference: −1 nmol/L; *p* = 0.82) resulting from a significant reduction in small VLDL-P (−5 nmol/L; *p* = 0.03; Table 3). An increase in VLDL particle size was also seen with colesevelam (*p* = 0.001; Fig. 1).

Treatment with colesevelam relative to placebo was also associated with a nonsignificant increase in total HDL-P (0.6 μmol/L; *p* = 0.20) attributable to significant increases in large (0.5 μmol/L; *p* = 0.007) and medium (0.8 μmol/L; *p* = 0.02) HDL-P (Fig. 1). HDL particle size was also increased relative to placebo in subjects treated with colesevelam (*p* < 0.0001; Fig. 1).

Subjects who received colesevelam, compared with placebo, also had statistically significant percent changes from baseline in total LDL-P (LS mean percent treatment difference: −10 %; *p* < 0.0001), small VLDL-P (−13 %; *p* < 0.05), and medium HDL-P (167 %; *p* = 0.002). The percent changes were not significantly different between colesevelam and placebo for the other particle classes.

### Safety

Overall, 16 (9.4 %) and 33 (18.9 %) subjects in the placebo and colesevelam groups, respectively, had an adverse event (AE) considered related to study medication. Hypoglycemia occurred at a higher incidence in the colesevelam treatment group compared with placebo (4.0 % vs. 0.6 %) and was generally mild overall. The majority of AEs were mild or moderate in severity. As reported in the primary study publication [22], a serious AE was reported by 4.0 % and 1.8 % of subjects in the colesevelam and placebo groups, respectively, and none were considered by investigators to be related to study medication. The most frequently reported AEs were back pain (5.1 %) and headache (4.6 %) in the colesevelam group and urinary tract infection (8.8 %) and nasopharyngitis (5.3 %) in the placebo group. There were no clinically important differences observed between the colesevelam and placebo groups in safety laboratory parameters.

## Discussion

This study in adults with T2DM of relatively short duration (mean 4.1 years overall) demonstrated generally favorable changes to the lipoprotein particle profile among those who received colesevelam as an antidiabetes monotherapy, compared with those who received placebo. Significant effects were seen on LDL particles (a reduction in total LDL-P largely attributable to reductions in small and very small LDL-P, as well as large LDL-P), VLDL particles (an increase in VLDL particle size with an accompanying reduction in small VLDL-P), and HDL particles (an increase in HDL particle size with accompanying increases in large and medium HDL-P).

It is increasingly recognized that the lipoprotein profile affects CVD risk, although the influence of lipoprotein size as an independent predictor of CVD risk is less clear than that of lipoprotein concentration [5, 11, 25–27]. LDL-P is predictive for coronary endothelial dysfunction and CVD risk [5, 6]. In one study, LDL-P was the strongest predictor of future CVD events of the lipoprotein parameters determined by NMR, with the risk for subsequent CVD events among subjects in the highest quartile of LDL-P being >4-fold that of the risk among subjects in the lowest quartile [5]. LDL-P remained significant even after adjusting for other lipid parameters, suggesting that LDL-P provides additional risk data beyond that provided by traditional lipoprotein parameters. Increased HDL-P and large HDL have also been shown to be associated with a reduction in CVD [6, 10, 25].

Importantly, LDL-P may be a better predictor of CVD than LDL cholesterol [4, 7–9]. Many patients with increased cardiometabolic risk have relatively normal LDL cholesterol levels but have abnormal distributions of lipoprotein particles [4, 9]. Thus, LDL cholesterol incompletely measures the risk for coronary artery disease (CAD) [6, 8, 9, 28]. In particular, since many patients with metabolic syndrome have a relatively normal LDL cholesterol level but an elevated LDL-P, traditional monitoring of LDL cholesterol may underestimate CVD risk [28]. Our finding that colesevelam reduced LDL cholesterol levels and LDL-P (primarily through reductions in small and very small LDL-P) reflects a shift to a less atherogenic profile. Small LDL particles have been shown in several studies to be predictive of CVD events and CAD [11, 29, 30].

These results are in line with previous studies showing that colesevelam decreases LDL-P and increases LDL particle size in subjects with hypercholesterolemia [31], prediabetes [32], or T2DM [20, 21]. In the previous studies in subjects with T2DM [20, 21], as in the current study, the reduction in LDL-P was largely attributable to reductions in the smaller LDL particle subclasses: small LDL-P, as well as very small LDL-P (assessed in the current study). The reduction in very small LDL-P with colesevelam is notable given that such an effect is not seen with all other lipid-lowering agents, with some even showing an increase in this parameter [33, 34].

Despite the increase in large VLDL-P, colesevelam reduced glucose levels, which suggests that the increase in large VLDL-P does not result from worsened insulin resistance. There was also a lack of correlation between reductions in LDL-P and reductions in A1C or FPG. This may reflect the current understanding of colesevelam’s independent mechanisms of LDL cholesterol lowering (via deactivation of the farnesoid X receptor in the intestine) and glucose lowering (via stimulation of TGR5 in the colon, secretion of the incretin hormone glucagon-like peptide-1, and reduced hepatic glucose production through suppression of glycogenolysis) [35].

A strength of this study is that the observed lipid effects can be directly attributed to colesevelam since only a small proportion of subjects administered colesevelam was on preexisting statin therapy. However, the small portion of subjects on statin therapy also limits the ability to analyze cohorts based on the use of a statin in combination with colesevelam versus colesevelam therapy alone. Consequently, it would be difficult to extrapolate the study results to determine the effect of colesevelam in patients with T2DM and elevated LDL-cholesterol levels who were already taking a statin. However, it is noteworthy that a pooled analysis of data from three pivotal studies in patients with T2DM showed that adding colesevelam to concomitant statin therapy significantly reduced LDL cholesterol by an additional 16 % compared with a 1 % reduction in patients receiving placebo plus concomitant statin therapy (*p* < 0.0001) [36]. In addition, compared with previous studies assessing the lipoprotein particle profile, subjects in the present study were not taking other antihyperglycemic medications such as metformin and/or sulfonylurea as they had in the previous studies [20, 21]. Thus, the effect of colesevelam on the lipid profile described in the present study would not have been influenced by prior or concomitant exposure to other antidiabetes agents.

## Conclusions

In subjects with T2DM, colesevelam as an antidiabetes monotherapy produced generally favorable changes in the lipoprotein particle profile compared with placebo.
